# Design of Liquid Formulation Based on F127-Loaded Natural Dimeric Flavonoids as a New Perspective Treatment for Leishmaniasis

**DOI:** 10.3390/pharmaceutics16020252

**Published:** 2024-02-08

**Authors:** Camila Silva da Costa, Estela Mesquita Marques, Jessyane Rodrigues do Nascimento, Victor Antônio Silva Lima, Ralph Santos-Oliveira, Aline Santana Figueredo, Caroline Martins de Jesus, Glécilla Colombelli de Souza Nunes, Clenilma Marques Brandão, Edson Tobias de Jesus, Mayara Coelho Sa, Auro Atsushi Tanaka, Gustavo Braga, Ana Caroline Ferreira Santos, Roberto Batista de Lima, Lucilene Amorim Silva, Luciana Magalhães Rebelo Alencar, Cláudia Quintino da Rocha, Renato Sonchini Gonçalves

**Affiliations:** 1 Laboratory of Chemistry of Natural Products, Department of Chemistry, Federal University of Maranhão, São Luís 65080-805, Brazil; cs.costa@discente.ufma.br (C.S.d.C.); estelamarques.adv@gmail.com (E.M.M.); jessyanernascimento@gmail.com (J.R.d.N.); vas.lima@discente.ufma.br (V.A.S.L.); rocha.claudia@ufma.br (C.Q.d.R.); 2 Postgraduate Program in Chemistry, Institute of Chemistry, UNESP-Estadual University Paulista Júlio de Mesquita Filho, Araraquara 14800-060, Brazil; 3 Nuclear Engineering Institute, Brazilian Nuclear Energy Commission, Rio de Janeiro 21941-906, Brazil; presidenciaradiofarmacia@gmail.com; 4 Laboratory of Nanoradiopharmacy, Rio de Janeiro State University, Rio de Janeiro 23070-200, Brazil; 5 Laboratory of Immunophysiology, Center for Biological and Health Sciences, Federal University of Maranhão, São Luís 65080-805, Brazil; aline.sf@discente.ufma.br (A.S.F.); caroline.mj@discente.ufma.br (C.M.d.J.); lucilene.silva@ufma.br (L.A.S.); 6 Research Nucleus in Pharmaceutical Sciences Program, State University of Maringá, Maringá 87020-900, Brazil; profglecillacolombelli@gmail.com; 7 Department of Chemistry, Federal Institute of Maranhão, São Luis 65075-441, Brazil; clenilma.brandao@ifma.edu.br (C.M.B.); tobiasedson@ifma.edu.br (E.T.d.J.); mayara.sa@ifma.edu.br (M.C.S.); 8 Department of Chemistry, Federal University of Maranhão, São Luís 65080-805, Brazil; tanaka.auro@ufma.br (A.A.T.); gustavo.braga@ufma.br (G.B.); anacaroline.ufma@yahoo.com.br (A.C.F.S.); rb.lima@ufma.br (R.B.d.L.); 9 Laboratory of Biophysics and Nanosystems, Department of Physics, Federal University of Maranhão, São Luís 65080-805, Brazil; luciana.alencar@ufma.br

**Keywords:** leishmaniasis, *Arrabidaea brachypoda*, pluronic F127

## Abstract

Infectious and Parasitic Diseases (IPD) remain a challenge for medicine due to several interconnected reasons, such as antimicrobial resistance (AMR). American tegumentary leishmaniasis (ATL) is an overlooked IPD causing persistent skin ulcers that are challenging to heal, resulting in disfiguring scars. Moreover, it has the potential to extend from the skin to the mucous membranes of the nose, mouth, and throat in both humans and various animals. Given the limited effectiveness and AMR of current drugs, the exploration of new substances has emerged as a promising alternative for ATL treatment. *Arrabidaea brachypoda* (DC). Bureau is a native Brazilian plant rich in dimeric flavonoids, including Brachydin (BRA), which displays antimicrobial activity, but still little has been explored regarding the development of therapeutic formulations. In this work, we present the design of a low-cost liquid formulation based on the use of Pluronic F127 for encapsulation of high BRA concentration (LF-B500). The characterization techniques revealed that BRA-loaded F127 micelles are well-stabilized in an unusual worm-like form. The in vitro cytotoxicity assay demonstrated that LF-B500 was non-toxic to macrophages but efficient in the inactivation of forms of *Leishmania amazonensis* promastigotes with IC_50_ of 16.06 µg/mL. The results demonstrated that LF-B500 opened a new perspective on the use of liquid formulation-based natural products for ATL treatment.

## 1. Introduction

In the 21st century, Infectious and Parasitic Diseases (IPD) remain a challenge to the scientific community, even considering the numerous prophylactic strategies and antimicrobial drugs developed in recent years [1,2]. IPD are mostly endemic diseases, especially in working-class regions of underdeveloped and developing countries, and have become a significant public health problem. The complexity of combating IPD is closely associated with factors intrinsic to the sick individual, such as nutritional status, immunological aspects, pre-existing diseases, and social, cultural, and environmental factors [3]. ATL is a neglected IPD that gives rise to difficult-to-treat skin sores, leading to severe scarring. Additionally, it can extend from the skin to the mucous membranes of the nose, mouth, and throat in both humans and various animals [4,5]. Several species of digenetic protozoa of the order Kinetoplastida, family Trypanosomatidae, cause ATL. The genera *Leishmania* (*Viannia*) *braziliensis*, *Leishmania* (*Leishmania*) *amazonensis*, and *Leishmania (Viannia) guyanensis* are mainly responsible for human ATL in Brazil [6]. The first-line drugs employed for the treatment of leishmaniasis are the pentavalent antimonials, specifically meglumine antimoniate (Glucantime) and sodium stibogluconate (Pentostam). Additional pharmaceutical options may encompass pentamidine and amphotericin B [7]. Miltefosine is an oral medication that has been recently approved for the treatment of ATL [8,9]. It works by interfering with the parasite’s cell membrane and is effective in both cutaneous and mucocutaneous leishmaniasis [10]. However, the unavoidable damage to the adjacent normal tissue upon treatment at high doses or prolonged use of these medicines can cause more severe side effects, such as liver and kidney damage, cardiac arrhythmias, and pancreatitis, as well as antimicrobial resistance (AMR) to the drugs [11]. In addition to medication, wound care is an important aspect of ATL treatment. Proper wound care can help prevent secondary infections and promote healing. In some cases, surgery may be necessary to remove large ulcers or lesions [12]. Due to this limited therapeutic efficacy, AMD, and the high toxicity of available drugs, the search for new substances inspired by empirical methods has become a promising alternative for ATL [13]. Fortunately, the reservoir of medicinal flora houses biologically active compounds endowed with therapeutic properties, extensively documented and employed by diverse communities for addressing a spectrum of ailments [14]. As per the Brazilian Fund for Biodiversity (FUNBIO), the botanical wealth in Brazil encompasses around 55,000 described species, constituting 22% of the global total [15]. Within arid forests akin to those witnessed in the Cerrado, plant species manifest a profusion of polyphenolic compounds, notably featuring flavonoids, which have been the subject of considerable scientific interest in the fight against neglected diseases [16,17,18]. In recent years, our research group has published several papers uncovering the medicinal properties of the unusual dimeric flavonoids, *Brachydin* (BRA), extracted from *Arrabidaea brachypoda* (DC) Bureau, a native Brazilian plant used in folk medicine against a range of ailments, including respiratory problems, inflammation, and pain [19]. The potential cytotoxic effect of these dimeric flavonoids, against both promastigotes and amastigotes forms, opens a perspective for developing new efficacious formulations for leishmaniasis treatments [20,21]. In previous work, Rocha et al. studied the leishmanicidal activity of isolated BRA against the amastigote form. Ultra-structural analysis showed the ability of the BRA to induce cell lesions, which progressed to parasitic death. The observed Golgi damage, concurrent with the accumulation of vesicles within the flagellar pocket, signifies a disruption in endocytic pathways within amastigotes. This vesicle accumulation potentially denotes an aberration in the release mechanism or an upsurge in both vesicle production and exocytosis. Notably, the vesicular dynamics within amastigotes play a pivotal role in nutrient acquisition, intracellular communication, virulence modulation, and the intricate interplay between host and pathogen [22].

In this paper, we have designed a low-cost liquid formulation based on Pluronic F127 loaded with high BRA concentration. F127 is a non-ionic surfactant approved by the U.S. Food and Drug Administration (FDA) for use as an excipient in various pharmaceutical and biomedical applications. F127 is an ABA triblock copolymer comprised of poly(ethylene oxide) (EO) and poly(propylene oxide) (PO) blocks, which self-assemble in core-shell micelles above the critical micelle concentration (CMC) in an aqueous medium [23]. Using F127 copolymer as a drug delivery system allows the encapsulation of poorly water-soluble drugs, enhancing their bioavailability, providing controlled release over time, better cellular uptake, and a prolonged drug release effect on the therapeutic target [24,25]. Liquid formulations have garnered extensive utilization in the field of pharmaceutics owing to their heightened dosing flexibility, ease of ingestion, and rapid onset of therapeutic effects [26]. In the case of monophasic liquid, where the active pharmaceutical ingredient is completely dissolved within the vehicle, the liquid dosage forms are oral, parenteral, and external, or special, uses [27]. The high encapsulation efficiency of the active pharmaceutical ingredient (API) on nanoformulation allows a reduction in the administered dose and, consequently, reduces adverse reactions when compared to conventional formulations [28]. In this work, a liquid formulation based on an F127 copolymer loaded with BRA was developed as an alternative for the oral treatment of ATL.

## 2. Materials and Methods

Pluronic F127 (poly(ethylene oxide)–poly(propylene oxide)–poly(ethylene oxide)) triblock copolymer (Mw = 12,600 g·mol^−1^; (EO)_99_(PO)_67_(EO)_99_), solvents of HPLC grade dichloromethane (DCM), methanol (MeOH), ethyl alcohol (EtOH), acetonitrile, formic acid, and ultrapure water), Millex^®^-GP syringe filter, phosphate buffer, fetal bovine serum (FBS), and 3-(4,5-dimethyithiazol2-yl))-diphenyl tetrazolium bromide (MTT) were commercially acquired from the Merck company (Rahway, NJ, USA).

### 2.1. Extraction, Isolation, and Characterization of BRA

**Scheme 1:**
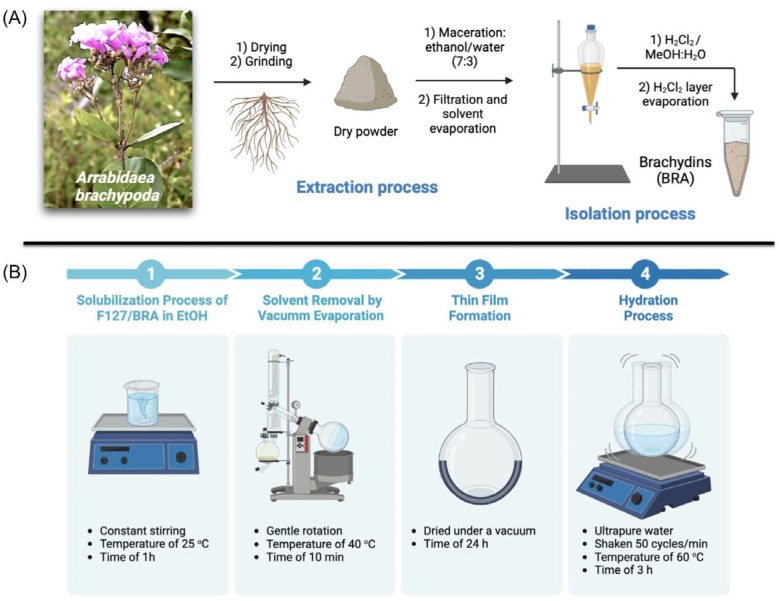
Schematic experimental steps for (**A**) extraction and isolation of BRA from *Arrabidaea brachypoda* roots and (**B**) preparation of BRA-loaded F127 micelles by the solid dispersion method.

Roots of *Arrabidaea brachypoda* were collected in April 2021 at Sant’Ana da Serra farm, João Pinheiro, Minas Gerais, Brazil. Plant identification was conducted at the ICEB of the José Badine Herbarium of the Federal University of Ouro Preto by Professor Maria Cristina Teixeira Braga Messias. A voucher specimen (no. 17935) was deposited at the Herbarium of the Federal University of Ouro Preto, Brazil. Collection activities were carried out following Brazilian laws on the protection of biodiversity (SisGen No. A451DE4). The extraction and fractionation procedures followed previously described methods [24]. Briefly, *Arrabidaea brachiopoda* roots (300 g) were dried and ground in a knife mill. The resulting powder was then macerated with ethanol/water (7:3). After filtration, the ethanolic extract of the roots was collected and evaporated under reduced pressure (40 °C), resulting in 11.8 g of an aqueous-ethanol extract. This extract was subsequently diluted in H_2_O and subjected to liquid–liquid partitioning with dichloromethane (DCM) and methanol to obtain dichloromethane and methanol/water extracts, respectively. Further liquid–liquid extractions on the aqueous-ethanol extract were performed using DCM (1 L) and H_2_O−MeOH (7:3) (1 L). The crude DCM and aqueous-methanolic fractions were obtained after decantation and evaporated under a vacuum at approximately 40 °C. The DCM was evaporated by rotary evaporation at 40 °C to yield 37.7% (4.44 g) of a mixture of BRA as a brown solid (Scheme 1A). The DCM fraction enriched with BRA was characterized through a Shimadzu model HPLC system (Shimadzu Corp., Kyoto, Japan), incorporating a binary pump module, a UV-PDA detector set at 254 nm, and a mass spectrometer. The analysis was conducted on a 5 μm C18 100 A L column (150 μm × 4.6 μm). The mobile phase consisted of a gradient mixture of water and methanol acidified with 0.01% formic acid, with a flow rate of 1 mL/min. The gradient elution ranged from 70% to 100% methanol over 70 min, and another 10 min of 100% methanol (Figure S1).

### 2.2. Nanoencapsulation Method

The encapsulation of BRA on F127 nanostructured micelles was carried out by the solid dispersion method following Scheme 1B [28]. F127 copolymer was dissolved in ethanol and the solution was maintained under constant stirring at 25 °C for 30 min. Subsequently, a solution containing BRA in ethanol was introduced and kept under continuous stirring at 25 °C for 30 min. The solvent was then removed through rotary evaporation at 40 °C, resulting in a thin film that underwent vacuum drying for 24 h to eliminate any residual solvent traces. This dried thin film was rehydrated with ultrapure water and subjected to shaking in a Dubnoff shaking water bath (Thermo Scientific Precision DUB 15 L (Waltham, MA, USA)) at 50 cycles per minute, maintaining a temperature of 60 °C for 3 h. This process aimed to obtain BRA-loaded F127 micelles at concentrations of 0.5%, 0.25%, or 0.125% (*w*/*v*) in F127, with a BRA concentration of 500 μg/mL. The F127 micelles were consistently prepared as controls for all experiments.

### 2.3. FTIR Characterization

The F127 and BRA-loaded F127 micelles were characterized by reflectance–Fourier transform infrared spectroscopy (FTIR) to prove the encapsulation of BRA on F127 nanostructured micelles. A Tracer-100 FTIR spectrophotometer (Shimadzu) was used to acquire all MIR spectroscopic data. The data acquisition was performed within a spectral range of 400–4000 ν under Happ–Genzel apodization. The analysis was performed for BRA, F127, and BRA-loaded F127 lyophilized formulations in KBr pellet form and the spectra were collected at room temperature with 50 scans and 8 cm^−1^ spectral resolution.

### 2.4. Encapsulation Efficiency (EE%)

The *EE*% assessment was conducted through high-performance liquid chromatography coupled with a UV detector (HPLC-UV), employing a Shimadzu model HPLC system (Shimadzu Corp., Kyoto, Japan). The system comprised a solvent injection module with a binary pump and UV-VIS detector (SPA-10A). The column utilized was a Luna 5 µm, C18 100 Å (250 μm × 4.6 μm). Elution solvents included A (2% formic acid in water) and B (2% formic acid in methanol), with a flow rate of 1.0 mL/min and a methanol gradient of 70–75% in 0–10 min, 75–100% in 10–20 min, and 100% in the subsequent 10 min. The injection volume was 10.0 μL. Data acquisition and processing were performed using LC Solution software version 2.3 (Shimadzu).

### 2.5. Accelerated Stability Testing

To investigate the influence of temperature on the physical and chemical stability properties of the BRA-loaded F127 micelles, accelerated stability testing was performed according to the Cosmetics Stability Guide from ANVISA and US Pharmacopoeia [29,30]. One milliliter of the formulation was submitted to centrifugation at 3000 rpm for 30 min at 25 ± 1 °C. The formulation was stored throughout the study duration either at room temperature (25 ± 3 °C) or under controlled refrigeration (5 ± 3 °C) (temperature was monitored daily). Then, the formulation was submitted to 7 cycles of 24 h at 5 °C and 24 h at 25 °C. The chemical stability of BRA-loaded F127 micelles was evaluated by HPLC-UV analysis comparing the BRA concentration at the end of the seventh cycle with the BRA concentration determined by *EE*% assay (day 0) [20]. The physical stability was determined concerning homogeneity and phase separation, as well as by analysis of the organoleptic characteristics (appearance, color, and smell).

### 2.6. Atomic Force Microscopy (AFM)

**Figure 1:**
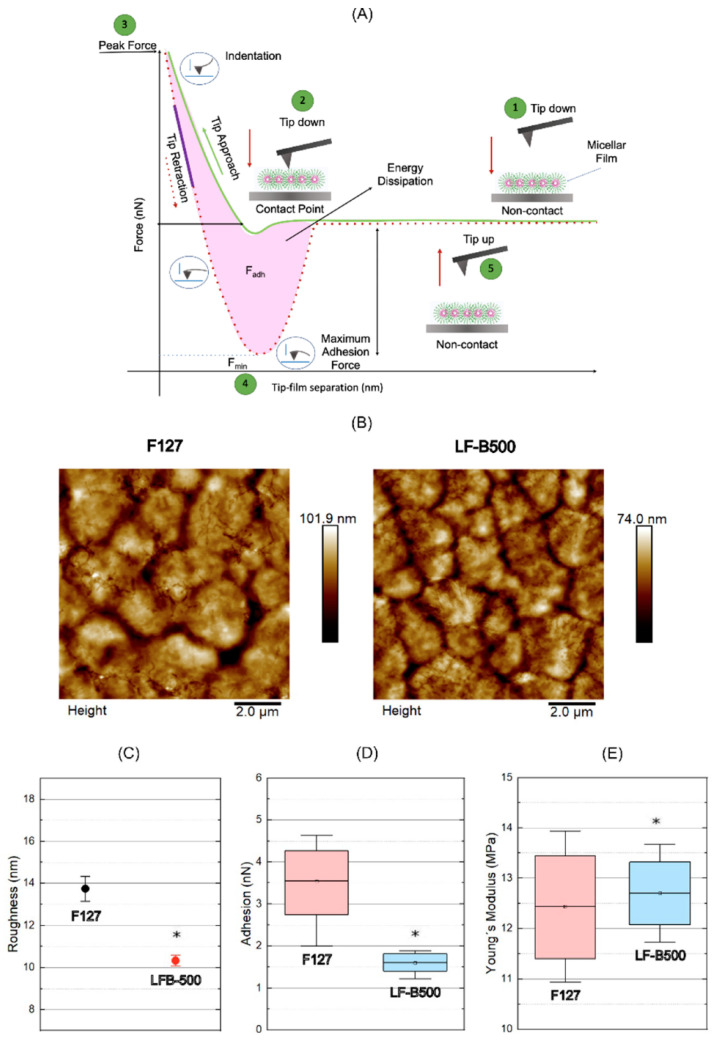
(**A**) Schematic diagram of a typical force versus separation curve obtained with AFM. (**B**) Topographic maps of the nanostructured micelles. (**C**) Root mean square of the roughness plot. The values found for the empty F127 micelles (F127). Significant value differences were determined by ANOVA statistics with post-test Tukey *p* < 0.05. (**D**) Graph of adhesion forces with mean values of 3.5 ± 0.9 nN and 1.6 ± 0.3 nN, respectively, for F127 and LF-B500. The inner image represents the behavior of the AFM probe during the film interaction in the analysis of adhesion forces. (**E**) Young’s modulus plot with mean values of 12.4 ± 1.4 MPa for the F127 and 12.8 ± 1.0 MPa for the LF-B500. The inner image represents the behavior of the AFM probe during the film interaction in the analysis of elastic forces. (*) Values with a significant difference in the ANOVA statistics with post-test Tukey *p* < 0.05.

The AFM analysis of the F127 and BRA-loaded F127 micelles was performed using a Multimode 8 microscope (Bruker, Santa Barbara, CA, USA). qpBioT (Nanosensor) probes were used with a nominal spring constant of 0.08 N/m. The thermal noise method was used to calibrate the actual spring constant of each probe. The F127 or BRA-loaded F127 micelles solution was applied onto freshly cleaved mica to generate a thin film. The scanning methodology employed was Peak Force Tapping Quantitative Nanomechanics (QNM), featuring a resolution of 256 × 256 lines per scan and a scanning frequency of 0.5 Hz. Topographic properties were observed, such as the films’ structure, size, height, and mean square roughness (Rq). The statistical roughness analysis was based on the height of each pixel in the image, analyzed from the height map. The parameter Rq is defined by Equation (1),

(1) $$\sqrt{\frac{1}{N}\sum_{i=1}^{N}\;\;z_{i}^{2}}$$

where *z* is each pixel height, and *N* is the total number of pixels (256 × 256), with scan size standardized to 10 × 10 µm. Before roughness analysis, maps were pretreated with a third-order polynomial fit. The diameters of the nanostructured films were measured using the Nanoscope Analysis 2.0 software with a Section Analysis tool, taking the width distance at the half-height of each structure, and the values were expressed as mean ± SD. Different samples’ adhesion (*F_adh_*) and Young’s modulus (*E*) were calculated from all force curves. The adhesion force between the probe and the sample surface was obtained from each AFM retraction curve (Figure 1A), considered at the minimum cantilever deflection value (*F_adh_*). This value represents the AFM probe’s resistance to leaving the sample surface. The value of *E* is obtained by fitting the retract curve using the DMT model given by Equation (2),

(2) $$F_{tip}=\frac{4}{3}E\sqrt{Rd^{3}}+F_{adh}$$

where *F_tip_* represents the force exerted on the tip, *R* denotes the radius of the tip end, and *d* signifies the separation between the tip and sample.

### 2.7. Scanning Electron Microscope (SEM)

The morphological characterization of BRA-loaded F127 micelles was conducted using SEM. The samples were frozen in liquid nitrogen and subsequently lyophilized in a Thermo Micro Modulyo freeze dryer (Thermo Electron Corporation, Pittsburgh, PA, USA). Following this, the lyophilized samples were subjected to metallization using a BAL-TEC Sputter Coater (model SCD 050, Balzers, Liechtenstein), and their morphology was assessed at magnifications of 100 and 50 times in an FEI Quanta 250 microscope (Thermo Fisher Scientific, Karlsruhe, Germany).

### 2.8. Particle Size and ζ Potential

The particle size and zeta (ζ) potential analyses of the nanoformulations were performed by dynamic light scattering (DLS) technique using a Litesizer™ 500 (Anton Paar GmbH, Graz, Austria) instrument (Module BM 10). Average hydrodynamic diameters (Dh) and ζ potential analyses of F127 and BRA-loaded F127 were determined in water at 25 °C and a 40-mW semiconductor laser of 658 nm. The Dh measurement was performed using a quartz cuvette of 3.0 mL. The ζ potential was performed using a low-volume cuvette (Univette). All the measurements were performed in triplicate (mean ± SD).

### 2.9. Oxidation Stability Assay

Electrochemical analyses were performed using an Autolab PGSTAT 302N potentiostat, Netherlands, coupled to a microcomputer and controlled by the GPES 4.9 program (General Purpose Electrochemical Software), used for potential control and data acquisition. A three-electrode cell was used in all experiments, the reference electrode Ag/AgCl (saturated KCl), the spiral platinum electrode as a counter, and the glassy carbon electrode (GCE) as a working electrode. Before each measurement, the GCE was polished with aluminum slurry and sonicated in deionized water for 5 min. The electrochemical analysis system consisted of a cell with 4 mL of 0.1 mol L^−1^ phosphate buffer, pH 5.5, with a potential range varying from 0 to 1.50 V vs. Ag/AgCl.

### 2.10. Cytotoxicity Assay on Macrophages

RAW 264.7 murine macrophages (donated by the Applied Immunology Laboratory, Rio de Janeiro, Brazil) were kept in sterile culture bottles with RPMI 1640 medium (SIGMA, Livonia, MI, USA) containing penicillin (100 µg/mL), streptomycin (100 U/mL), amphotericin B (0.25 µg/mL) and supplemented with 5% fetal bovine serum (FBS) in a 5% CO_2_ oven at 37 °C. For the cytotoxicity assay, 2 × 10^6^/mL of cells were used in RPMI-1640 medium supplemented with 1% SFB. The cells were added in a volume of 100 µL per well in 96-well flat bottom plates. The macrophages were then placed in a 5% CO_2_ oven for 1 h to adhere to the bottom of the plate. Next, the macrophages were incubated with different concentrations of the F127 and BRA-loaded F127 micelles (500–1.9 µg/mL) to calculate the cytotoxic concentration of 50% of the cells (CC_50_). Untreated cells (negative control) and cells treated with concentrated DMSO (positive control) were kept as controls. After incubation for 48 h, MTT (5 mg/mL) was added to the culture, followed by a new incubation (3 h, 37 °C). Cell viability was assessed based on MTT metabolism, which was proportional to the absorbance value generated in a spectrophotometer at a wavelength of 540 nm. After the incubation time, 100 µL of DMSO per well was added and read in a 540 nm Elisa reader, and the percentage of cytotoxicity was calculated (% cytotoxicity = (1 − Optical density of the test/Average of the negative control) × 100) [26]. Cytotoxicity was expressed as a percentage, and CC_50_ was determined using the GraphPad Prism 8 program.

### 2.11. Cytotoxicity Assay on Leishmania Amazonensis Promastigotes

The anti-Leishmania effect of the F127 and BRA-loaded F127 micelles was studied on promastigote forms of *L. (L.) amazonensis* (MHOM/Br/90/BA125) in the stationary growth phase (4 days of culture) 5 × 10^7^/mL–10 µL, which were added to 96-well plates (5 × 10^5^ mL per well) in the presence of different concentrations (500–1.9 µg/mL). Pentamidine^®^ (IC_50_ = 4 µg/mL) was used as a positive control and the negative control was prepared with Schneider’s culture medium only. After the incubation time (48 h), 10 µL of MTT (5mg/mL) was added and incubated again for 3 h to allow the formation of formazan crystals. Finally, 100 µL of 10% DMSO was added, and the absorbance was accessed in a spectrophotometer at 570 nm after 24 h. The 50% inhibitory concentration (IC_50_) was obtained by non-linear regression using GraphPad Prism version 8.0.2.

### 2.12. Statistical Analysis

The evaluation of normal distribution, based on a single criterion, was carried out through analysis of variance (ANOVA) and Tukey’s post-test, with statistical significance determined at *p* < 0.05. Statistical analyses and graphical representations were executed using ORIGIN software é: 10.0. The calculated error for all data was presented as the standard deviation (mean ± SD).

## 3. Results and Discussion

### 3.1. Preliminary Stability Assay

**Scheme 2:**
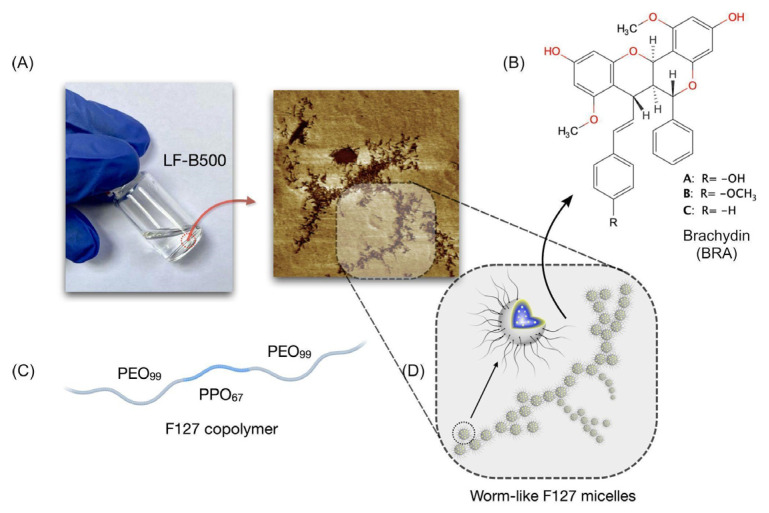
(**A**) Picture of the liquid formulation LF-B500 showing physical stability after 72 h of preparation. (**B**) Chemical structure of brachy-dins A, B, and C. (**C**) Representation of the F127 copolymer composition. (**D**) Schematic representation of worm-like F127 micelles observed by morphology analysis.

The solid dispersion method was carried out to prepare copolymeric nanoparticles loaded with BRA. The structural integrity of the formulation was evaluated in a preliminary assay, by visual analysis. Firstly, the capacity of the F127 copolymeric nanoparticles on the drug solubilization in the hydration step was observed at room temperature for 0.125% (*w*/*v*) in F127 and 500 μg/mL of BRA. The visual analysis was followed by 72 h, during which the formulations were not stable, with loss of structural integrity due to the formation of filamentous precipitates. The subsequent essay evaluated the stability of micelles in the function of the increased concentration of F127. Thus, the F127 was set to 0.5% and 0.25% (*w*/*v*) for 500 μg/mL of BRA. The hydration step was performed at room temperature. After 72 h, physical change was observed, with the formation of filamentous precipitates. The third essay evaluated the temperature effect on the stability of the formulations. The concentrations for F127 and BRA were 0.25% (*w*/*v*) and 500 μg/mL, respectively, and the temperature was set to 60 °C. After 72 h, no physical change was observed for the formulation. According to the preliminary stability assay, we set this experimental condition for the preparation of liquid formulation, which was named LF-B500 (Scheme 2).

### 3.2. FTIR

The FTIR analysis was performed to confirm the encapsulation of BRA on F127 micelles (Figure S2). The frequencies and assignments of the FTIR absorption bands of neat and BRA-loaded F127 micelles (LF-B500) are summarized in Table 1.

Comparing the spectra of isolated BRA and BRA-loaded F127 micelles, it can be seen that the main absorption bands characteristic of BRA are also present in the spectrum of F127/BRA, which is the main evidence of the encapsulation of BRA in F127 micelles. The sharp band in 1612 cm^−1^ of neat BRA corresponding to C=C stretching of conjugate alkene (Ph-CH=CH-R) is observed at 1647 cm^−1^ in the LF-B500 spectrum. The absorption bands relative to C=C stretching and C-H bending of benzene rings (1469, 821, and 689 cm^−1^) are also present in the LF-B500 spectrum. When the BRA molecules are encapsulated in F127 micelles, some bands shifts toward a higher wavenumber, these observations suggesting that PO groups of F127 copolymer can interact with high-hydrophobic BRA and break down its hydrogen bonding and intermolecular associations, like π-π stacking. Upon the solubilization of BRA molecules on the core of F127, the π-electrons and the lone pair on the oxygen atoms become available, leading the absorption bands to a blue shift (Δ_ν_ = 29 to 35 cm^−1^). On the other hand, the broad-band observed in the F127 spectrum at 655 cm^−1^ and attributed to the out-of-plane O-H bending vibration becomes sharper and shifts to 632 cm^−1^ on the spectrum of LF-B500. The blue shift of O-H vibration bending is probably due to the intermolecular hydrogen bonding of EO groups when the F127 micelles are loading BRA molecules, resulting in well-stabilized aggregate forms. The absorption band at 1629 cm^−1^ corresponding to hydrogen-bonded O-H stretching supported this evidence. We infer that the strong interaction between the EO terminal units gives rise to worm-like micelles, as observed by AFM and SEM analyses.

### 3.3. Accelerated Stability Testing

Following the guidelines set forth by the United States Pharmacopeia (USP), the evaluation of the stability of active pharmaceutical ingredients (APIs) involves a thorough examination of both chemical and physical stability, alongside sustained performance over time. It is essential to highlight that any physical changes in the dosage form should be easily reversible, such as through shaking, before dosing or administration [31]. Regarding chemical stability, preparations compounded in accordance with USP standards are considered stable if the concentration of the API remains within the range 90–110% of the initial value (day 0) [23]. Accelerated stability testing consists of the use of extreme temperature conditions to accelerate possible instabilities of the formulation LF-B500, which was submitted to cycles of 24 h at room temperature (25 ± 4 °C), or under controlled refrigeration (5 ± 3 °C). Regarding chemical stability, no significant degradation of BRA occurred in the LF-B500 formulation. Indeed, at the end of the seventh cycle, the concentration of BRA remained higher than 90%, meaning that the liquid formulation was chemically stable under regular storage temperatures (at 5 ± 3 °C or 25 ± 3 °C). Based on the examination of physical stability, no changes in color or odor were observed, and there were no instances of phase separation during the storage period. These results collectively confirm the stability, both physical and chemical, of the oral liquid dosage forms over a 14-day duration, regardless of the storage conditions.

### 3.4. EE%

A crucial aspect of nanoformulations is the quantity of API incorporated into the nanocarrier. This is commonly expressed as *EE*%, representing the percentage of API associated with the nanocarrier in relation to the total API in the system. The *EE*% assay was conducted to evaluate the capability of F127 nanoparticles to encapsulate BRA, following Equation (3),

(3) $$EE\%=\left(\frac{m_{total}-m_{free}}{m_{total}}\right)\times 100$$

where *m_total_* is the mass obtained from the total concentration of the drug, and *m_free_* is the amount of drug non-associated with the F127 micelles. Immediately after the LF-B500 preparation (day 0), the quantification of the BRA in the formulation was performed by HPLC-UV (Table S1 and Figure S3). The EE% determined for LF-B500 was 92.65 ± 0.48%, a high drug loading considering the poor water-soluble characteristic of BRA. From this result, it is possible that a low amount of F127 copolymer (0.125% *w*/*v*) can load BRA efficiently in a high concentration. With the high EE% of F127 micelles, the minimum amount of nanoparticles is needed to achieve the therapeutic level, which can reduce the potential adverse effect from overdosed materials, as demonstrated by in vitro cytotoxicity assay, and decrease the manufacturing cost of the LF-B500.

### 3.5. Morphological Characterization

AFM and SEM analysis were performed to access the morphology of the BRA-loaded F127 micelles. Figure 1B shows the topographic maps for the F127 and LF-B500 acquired by the AFM technique. For many scans in each group (n = 30), changes in topography are observed, leading us to the changes caused when F127 is loading BRA molecules. Thus, a root mean square quantify roughness analysis was performed for all maps in each group, and the results are shown in Figure 1C. Note that, for the F127, the mean square roughness Rq = 13.7 ± 1.8 nm (mean ± standard deviation), while for LF-B500, the Rq parameter decreases to 10.3 ± 0.7 nm. This result reveals how BRA affects the ultrastructure of the micelles, increasing its uniformity. Resolutions on the height scale (z-axis) of the atomic force microscope are extremely accurate, reaching the Angstrom scale [32], which makes this technique promising for structural evaluations in nanometric compounds [33]. Since the measurements were performed in the QNM (Quantitative Nanomechanics) mode, although sometimes the micellar structures are immersed in the polymeric film and are not visible in the topographic maps, the mechanical scanning mode allows us to evaluate structures underlying the analyzed surface [34].

**Figure 2:**
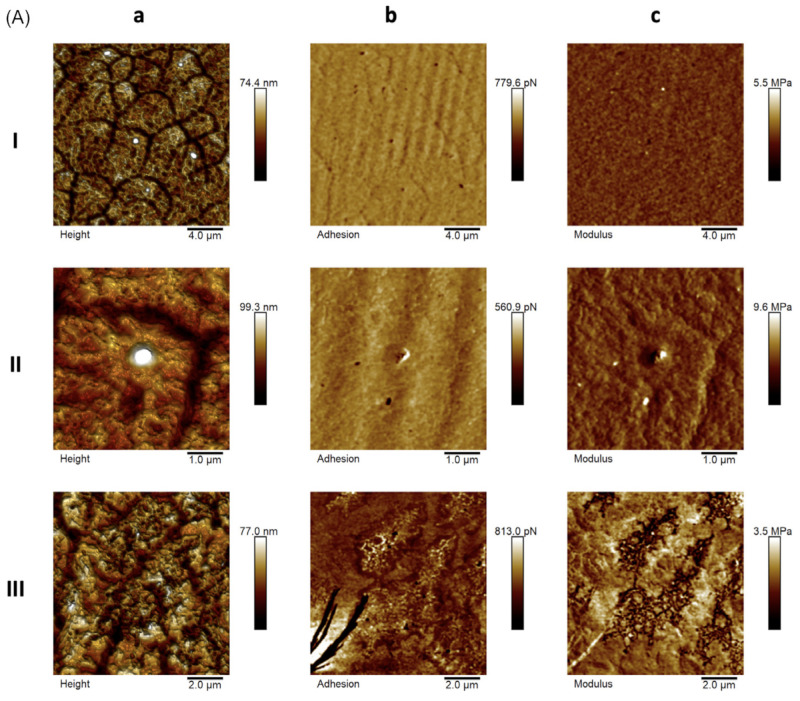

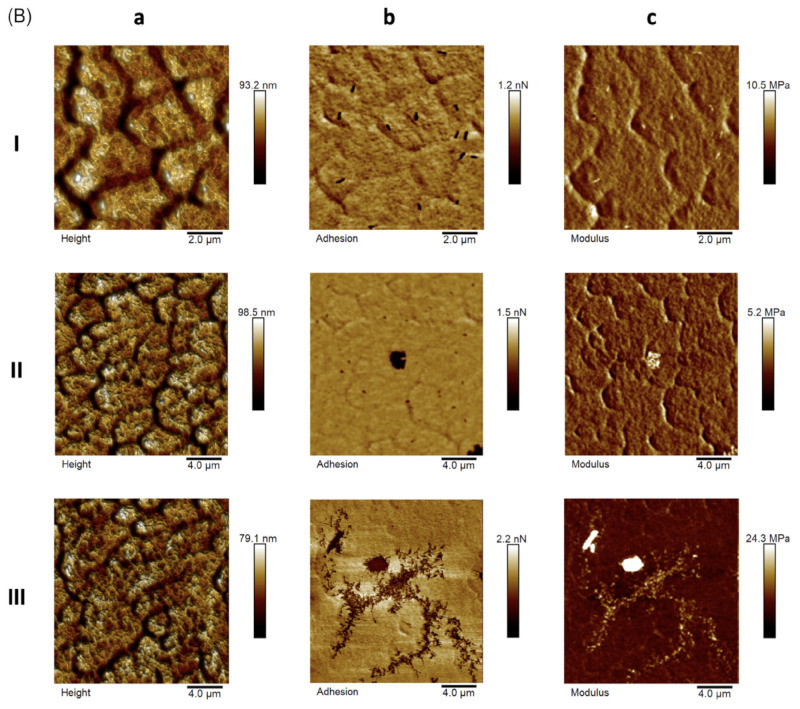
AFM representative images for (**A**) empty F127 micelles and (**B**) BRA-loaded F127 micelles: height (**aI**–**aIII**), adhesion force maps (**bI**–**bIII**), and Young’s modulus (**cI**–**cIII**).

**Figure 3:**
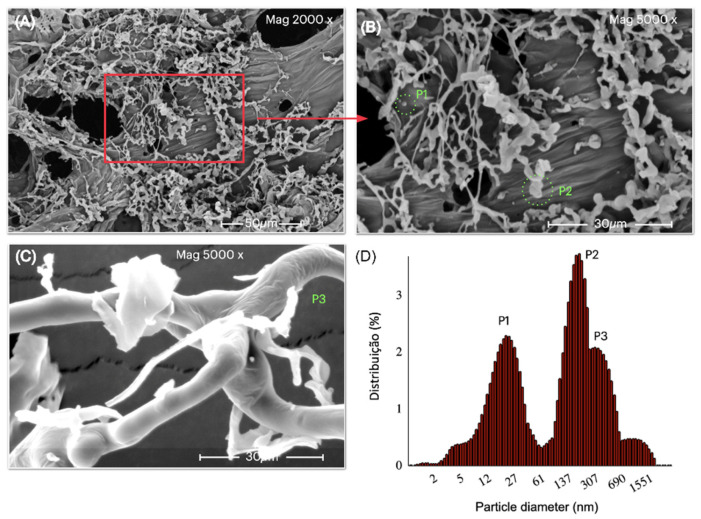
(**A**) SEM image of the LF-B500 formulation, (**B**,**C**) high-magnification SEM images, (**D**) DLS spectrum of LF-B500 formulation showing the particle size distribution in water solution. Mean size values for each peak and corresponding scattering intensity percentages are reported in Table 2.

Thus, it was possible to identify the presence of different micellar architectures (Figure 2A,B) in the maps of adhesion forces (bI–bIII) and Young’s modulus (cI–cIII). Considering the F127 copolymer concentration was set above the CMC for both formulations, micelles may become asymmetric, and eventually various aggregates may be formed. Such micellar aggregates may have the form of spherical objects, or they may be elongated stiff rods, less flexible worm-like, or more complex structures, such as branched worm-like [35,36,37,38,39,40,41]. The larger aggregates are interconnected by branches to form stable aggregates with even larger sizes, as shown in Figure 3D. Adhesion force mapping was determined for F127 and LF-B500. The adhesion maps are a combination of interaction responses between the AFM probe and the sample surface, but for high-frequency scans this response is mostly electrostatic [42]. In this way, in the darker region (lower values of *F_adh_*) shown in Figure 2A,B(bI–bIII), it is possible to identify the micellar architecture of the aggregates. Likewise, in Young’s modulus map shown in Figure 2A(cI–cIII), it can be seen that the aggregates formed by empty F127 micelles have higher *E* values when compared to the non-micellar region, inferring the stability of these nanostructures under the bulk of the F127 matrix. For the LF-B500, the BRA-loaded F127 micelles mostly result in larger aggregates, specifically in worm-like micelles, as shown in the adhesion maps Figure 2B (cI–cIII). Young’s modulus maps show higher *E* values in the center of the aggregates, inferring that the BRA molecules are preferably located in the more hydrophobic region (PO units) of the F127 micelles. Additionally, the lower values of *F_adh_* observed for the worm-like aggregates suggest the presence of negative charges. This result is according to negative *ζ* potential (−13 mV) measured by the DLS technique, which can infer that the smaller spherical micelles of diameter at about 20 nm can be stabilized as a larger aggregate of diameter at about 240 nm (see Table 2), resulting in a branched worm-like architecture. However, a statistic for the diameter of the micellar structures was calculated using the section analysis tool of the Gwyddion Software 2.6. The larger aggregates of LF-B500 have a mean diameter of 289.7 ± 11.3 nm.

These morphologies can also be evidenced by SEM analysis. Figure 3B,C shows the presence of small, interconnected spheres giving rise to worm-like aggregates.

Motivated by this unusual architecture found for BRA-loaded F127 micelles, and how it could affect the nanomechanical properties of LF-B500, we calculated the average values of adhesion forces and Young’s modulus, comparing them with the empty F127 micelles (Figure 1D). We observe that the mean value of *F_adh_* for the F127 is higher when compared to the LF-B500. The region of nanostructured micelles has lower adhesion forces (darker contrasts), which is a large statistic for scans, justifying the reduction of this mean value for the LF-B500. Furthermore, this is a nanomechanical marker of how BRA influences the biomechanical properties of worm-like aggregates. Mean ± SD values for samples F127 and LF-B500 are 3.5 ± 0.9 nN and 1.6 ± 0.3 nN, respectively. Young’s modulus values show how the insertion of BRA into the nanostructured micelles can influence the form of the non-usual aggregates as worm-like micelles (Figure 1E). There is an increase in the mean *E* of 12.4 ± 1.4 MPa for the F127 sample and 12.8 ± 1.0 MPa for the LF-B500, with a significant difference for the ANOVA test with the Tukey post-test. Although the increase is 3% of the global elasticity, the maps locally show that this result is probably related to the presence of BRA on F127 micelles.

### 3.6. Particle Size and ζ Potential

The particle size of nanostructured formulations was quantitatively measured using the DLS technique. The analysis of empty F127 micelles at 0.5% (*w*/*v*) and 25 °C shows different aggregates, with particle sizes of 16, 214, and 717 nm (Table 2). As observed by AFM, the morphologies of the aggregates of F127 can vary from isolated monomers to spherical micelles and large clusters, so-called worm-like micelles, which correspond to the most intense signals of scattered light. However, when the BRA was encapsulated on F127 micelles, the nanoparticle solution became slightly less polydisperse, and the architecture of the aggregates was changed. The peak associated with the large aggregates disappears, giving rise to a new peak centered at 247 nm with a highest relative abundance of 56%. The smaller architecture was also observed with a diameter of about 20 nm, probably associated with spherical micelles. Interestingly, as revealed by AFM, the spherical micelles can be physically interacting, forming branched worm-like micelles at about 350 nm. Worm-like micelles exhibit an overall length, known as the contour length, ranging from a few nanometers to micrometers [43]. The morphology of these micellar aggregates significantly influences their pharmacokinetics and biodistribution, resulting in remarkably prolonged blood circulation half-life times [44]. According to hypotheses derived from macrophage-capturing experiments, the unique shape of worm-like nanoparticles subjects them to a robust drag force from the flow, preventing macrophages from engulfing them before being carried away by the current [45].

The relative stability of the nanostructured micelles was evaluated by analyzing their ζ potential. The theory of ζ potential posits that the stability of a colloidal system is governed by the cumulative effect of van der Waals attractive forces and electrical double-layer repulsive forces. These forces come into play as particles approach each other, influenced by the Brownian motion they experience. Therefore, if the particles have a sufficiently high repulsion, the dispersion will resist flocculation and the colloidal system will be stable [46]. Regarding surface charge, achieving neutrality in nanoparticles could result in instability, leading to aggregation and precipitation over extended storage periods. Nevertheless, the characterization of surface charge is a vital parameter to assess in a nanoparticle suspension, since the initial interaction occurs with bodily fluids before reaching a target. Therefore, both formulations showed negative ζ potential (Table 2 and Figure S4). The negative values are in agreement with the maps of adhesion forces observed for empty and BRA-loaded F127 micelles, suggesting relatively high stability in the formation of the network aggregates in well-defined branched worm-like micelles (Figure 1D).

### 3.7. Oxidation Stability Assay 

**Figure 4:**
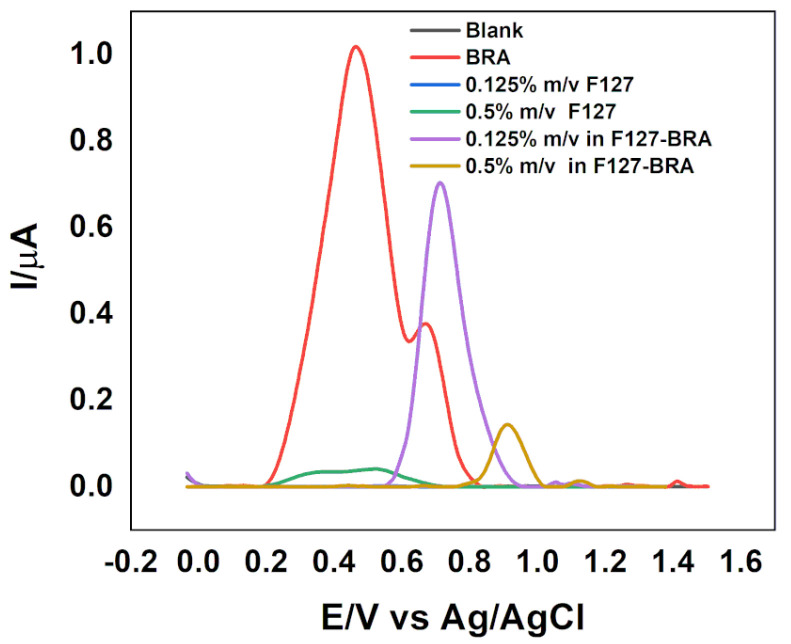
Differential pulse voltammograms on the GCE were recorded in different conditions: supporting electrolyte only (blank), in the presence of BRA, F127, and BRA-loaded F127 micelles in 0.1 mol L^−1^ phosphate buffer, pH 5.5, pulse amplitude = 50 mV and v = 25 mV s^−1^.

Electrochemical techniques are potentially useful tools used in the understanding of mechanisms, detection, characterization, and evaluation of antioxidant activity in plant extracts or synthesized samples, allowing us to relate their structure-activity with the effect on oxidation potential. In this context, BRA, F127, and Bra-loaded F127 micelles were studied. Figure 4 shows differential pulse voltammograms in the absence (Blank) and presence of BRA, F127, and BRA-loaded F127 micelles. In Blank, no Faradaic process is observed. For BRA, three main oxidation peaks were observed, according to the literature [47]; BRA-A presents a peak oxidation potential of around +0.48, while BRA-B and BRA-C present the oxidation process, the +0.71 and +0.57 V vs. Ag/AgCl, respectively.

**Figure 5:**
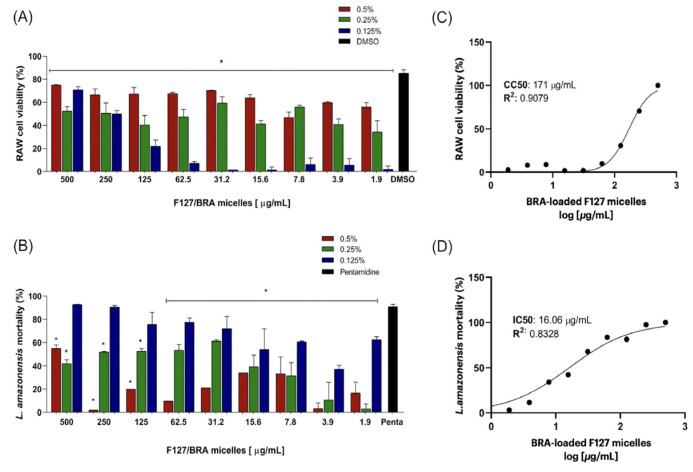
In vitro cytotoxicity assay of BRA-loaded F127 micelles against (**A**) RAW 264.7 cell line murine and (**B**) *Leishmania amazonensis* promastigotes. The assay was performed for different F127 copolymer concentrations (*w*/*v*): 0.5% (red bars), 0.25% (green bars), and 0.125% (blue bars), scanning a BRA concentration range of 500 to 1.9 µg/mL. The dose–response curves were accessed by linear regression for 0.125% *w*/*v* of the F127 copolymer. (**C**) 50% of RAW cell proliferation was achieved for a BRA concentration of 171 µg/mL, and (**D**) 50% of *Leishmania amazonensis* promastigotes proliferation was achieved for a BRA concentration of 16.06 µg/mL. The results correspond to (means ± SD) of individual samples tested in triplicate. (*) *p* < 0.05, compared to the positive controls (DMSO and pentamidine).

The F127 presents poorly defined oxidation peaks, with a small increase around +0.4 V vs. Ag/AgCl. The BRA-loaded F127 micelles resulted in an oxidation potential shift and a reduction in peak current, indicating that the encapsulation process made the oxidation of BRA more difficult. For 0.5% *w*/*v* in F127 loading 500 µg/mL of BRA, an impact on the electrochemical behavior was observed, and the peak current decreased significantly (0.14 µA), with a peak only around +0.91 V vs. Ag/AgCl. However, for 0.125% *w*/*v* in F127 loading 500 µg/mL of BRA, the potential shift is smaller (+0.72 V vs. Ag/AgCl). In addition, it presents a well-defined peak with a peak current of 0.7 µA, indicating that, at this concentration, we have a rapid charge transfer, suggesting rapid and unimpeded diffusion of ions when compared to the higher F127 concentration. These electrochemical aspects for BRA-loaded F127 micelles are affected by solubilization processes referring to the nature of the solute, the size of the core of these micelles, the concentration of the solute, extension of the hydrophilic corona, the polarity of the solute, hydrophobicity, charge, and degree of ionization, which also influence the solubilization capacity [48]. For micelles that contain incorporated hydrophobic API, the aggregation number (N) and Dh values are lower [49], and consequently changes in the interfacial area of the PEO blocks occur compared to the copolymer without the API [50]. Some studies report that the decrease in micellar size with the incorporation of APIs is linked to the greater hydrophobicity of the micelle in the presence of the encapsulated drug, meaning that fewer unimers of the copolymer are needed to form a micelle [51]. The F127 copolymer presents a hydrophilic balance in 70% of PEO groups [52], presenting more pronounced hydrophilic corona hydration, this being a tendency for copolymers that present a high degree of hydrophilicity due to the hydrogen bonds between the water molecules and the PEO groups [53]. This characteristic is largely due to the conformation that the copolymer assumes in solution, where the hydrophilic blocks (PEO) extend beyond the radius of the micelle, presenting a more fluid characteristic compared to more hydrophobic copolymers [54]. This fluidity explains why BRA-loaded F127 micelles with 0.125% *w*/*v* in F127 are more easily oxidized compared to 0.5% *w*/*v* in F127. This is reflected in the electrochemical results: once the same concentration of BRA is presented in F127 micelles, the 0.125% *w*/*v* in F127 has more BRA molecules per micelle, leading to more exposition of BRA to promastigote cells when compared to 0.5% *w*/*v* in F127, in agreement with the cytotoxicity assay (Figure 5). It can be inferred that the reason for 0.5% *w*/*v* in F127 loading 500 µg/mL of BRA is lower oxidation potential compared to 0.125% *w*/*v* in F127 loading 500 µg/mL of BRA. At a concentration of 0.5% (*w*/*v*), the PPO groups are less accessible to interaction with biological membranes, while at 0.125% (*w*/*v*) the micelles are more fluid, due to the greater interaction of the PEO groups with water molecules, which probably makes them more exposed as PPO groups, favoring their interaction with the promastigote cell membrane.

### 3.8. Leishmanicidal Activity

Pluronic F127 is an FDA-approved substance within the Pluronic family, known for its biocompatibility and utilization in living organisms. These compounds have undergone thorough research, confirming their safety for applications in both pharmaceutical and biomedical fields. Nevertheless, it is essential to acknowledge that, even though Pluronic F127 is FDA-approved and widely employed as an excipient in drug delivery applications, alterations in the formulation have the potential to modify the pharmacokinetics of an API, impacting systemic exposure. Therefore, the cytotoxicity of BRA and F127 copolymer was available by exposing the BRA-loaded F127 micelles to RAW 264.7 cell line murine. The assay was performed by setting three concentrations of F127 copolymer (0.5, 0.25, and 0.125% (*w*/*v*)) by decreasing the concentration of BRA 500 to 1.9 µg/mL, and the result was accessed by MTT assay (Figure 5A). For F127 concentrations of 0.5 and 0.25% (*w*/*v*), a significant variation in cell cytotoxicity of macrophages was observed even at the lowest concentrations of BRA. The mortality of RAW cells reached approximately 60% for a concentration of 0.5 and 0.25% (*w*/*v*) in F127 for a BRA concentration range of 7.8 to 1.9 µg/mL. These results indicate high toxicity of F127 on macrophages in these concentrations when compared to positive controls. However, a significant reduction in cytotoxicity was observed when the macrophage cells were exposed to 0.125% (*w*/*v*) in F127 copolymer. From 125 to 1.9 µg/mL of BRA, the cytotoxicity of the LF-B500 was reduced by about 20% to less than 5%, exhibiting a CC_50_ value of 171 (Figure 5C). It can be inferred that 0.125% (*w*/*v*) in F127 loading a relatively high BRA concentration (around 125 µg/mL) can be considered non-toxic for RAW cells and safety employed from a liquid formulation perspective.

The leishmanicidal potential of BRA-loaded F127 micelles was accessed by exposing the *Leishmania amazonensis* promastigotes to the same concentrations as the RAW cells. Interestingly, an inverse pattern for the viability of the promastigote forms was observed (Figure 5B). Even with the higher BRA concentrations, the mortality of promastigote forms did not reach 65% for the treatment with the LF-B500 at 0.5 and 0.25% (*w*/*v*) in F127. These results suggest that BRA concentration has a more significant influence on the mortality of promastigote forms, rather than in macrophages. On the other hand, the nanotoxicity of higher copolymer concentrations observed on the mortality of macrophages can be associated with its better ability to uptake the BRA-loaded F127 micelles. It is well known that the removal of some nanoparticles from circulation is thought to occur primarily through the phagocytic action of macrophages [55,56]. In this sense, considering the poor ability to uptake the nanoparticles by promastigote forms, the data on mortality appear to be more closely related to selectivity to BRA concentrations, i.e., to the relationship BRA/F127 micelle ratio, as observed also in previous works and inferred by oxidation assay [24,57,58]. The biological action of F127 Pluronic is more pronounced at lower concentrations of this copolymer, as they promote changes in the cell membrane. The hydrophobic portion composed of PPO groups immersed in the hydrophobic regions of the membrane results in changes in the morphology of the membrane, decreasing its viscosity (membrane fluidization). Contrasting the formation of micelles with a high concentration of poloxamers results in “hiding” the PPO groups in the hydrophobic core, decreasing the availability of the poloxamer to interact with cell membranes. The lipid environment of biological membranes is deeply related to the flow of drug transport [59]. The inhibition in the flow of drug transport to the extracellular environment occurs due to changes in the conformations of the proteins responsible for the flow, or steric hindrance in the interaction between drug-protein and specific binding site, induced by the copolymer chains immersed in the plasma of the cell membrane. The architecture of the hydrophobic core (PPO) and hydrophilic corona (PEO) structures is fundamental in the drug release process, where the micelles can be spherical, rod-shaped, worm-like (Figure 3 and Figure 4), or lamellar, depending on the size of the (PEO) and (PPO) groups, concentration and temperature [60]. The hydrophilic corona composed of PEO groups prevents aggregation and adsorption of proteins and prevents recognition by the reticuloendothelial system [61]. These characteristics make F127 extremely suitable for use in the development of formulations for encapsulating natural products, since it can be used in low concentrations to solubilize hydrophobic APIs, and mainly because it presents promising biological actions under these conditions. In this sense, the IC_50_ calculated for the BRA loaded in 0.125% (*w*/*v*) in F127 was 16.06 µg/mL, a good result compared to positive control (Figure 5D), whereas, for the 0.5 and 0.25% *w*/*v* in F127, the IC_50_ values were above 500 µg/mL. Comparing the CC_50_ results calculated for the lowest concentration of BRA-loaded F127 copolymer, a high selectivity index (SI = 10.64) is found for promastigote compared to RAW cells. This result highlights the leishmanicidal potential of the FB500 formulation and its low toxicity against macrophage cells. For the empty F127 micelle at 0.125% *w*/*v*, no significant toxicity was observed in RAW and promastigote forms above 250 µg/mL (Figure S5). However, complementary in vitro studies are being carried out to better understand the mechanisms of action of BRA-loaded F127 micelles under *Leishmania amazonensis* promastigote and amastigote forms and will be published in a forthcoming work.

## 4. Conclusions

This study involved formulating a liquid solution utilizing dimeric flavonoids extracted from *Arrabidaea brachypoda*, encapsulated within F127 copolymer micelles. Spectroscopic and morphological analyses indicated that the elevated concentration of BRA can be effectively stabilized within atypical worm-like F127 micelles. Results from the cytotoxicity assay demonstrated that BRA-loaded F127 micelles exhibit significant activity against Leishmania amazonensis promastigotes, while showing lower impact on macrophage cells. This discovery presents a promising new avenue for the treatment of ATL.

## Figures and Tables

**Table 1:** FTIR frequencies for neat BRA and BRA-loaded F127 micelles (LF-B500).

Compound Class	Wavenumber (cm^−1^)		Assignment
	BRA	LF-B500	
Methyl	2931	2966	symmetric C-H stretching
	2846	2875	anti-symmetric C-H stretching
	1369	1369	symmetric deformation
Alkene	1612	1647	C=C stretching
	960	-	CH wag (1,2-*trans*-disubstituted)
Benzene	1469	1469	C=C stretching
	821	871	C-H out-of-plane bending (mono-substituted benzene)
	698	698	C-H out-of-plane bending (1,4-substituted benzene)
Phenol	1269	1300	O-H bending
	1207	1249	C-C-O out-of-plane stretching
Ether	1107	1107	C-O-C stretching of aromatic ether
	1029	-	C-O-C stretching of cyclic ether

**Table 2:** Peak (P) ± SD, area (A), hydrodynamic diameter (Dh), polydispersity Index (PDI), and ζ potential of empty and BRA-loaded F127 micelles acquired by the DLS technique in triplicate at 25° C.

Formulation	P ± SD (nm) (A (%))			Dh (nm)	PDI (%)	ζ (mV)
F127	717 ± 300	214 ± 48	16 ± 5	796	30	−17
	(60)	(35)	(5)			
LF-B500	247 ± 100	23 ± 10	359 ± 119	157	28	−13
	(56)	(35)	(9)			

## Data Availability

The data presented in this study are available in this article.

## Supplementary Materials

The following supporting information can be downloaded at: https://www.mdpi.com/article/10.3390/pharmaceutics16020252/s1, Figure S1: Chromatographic profile of the DCM fraction of Arrabidaea brachypoda roots (λ = 254 nm), UV spectra, and chemical structure of the three dimeric flavonoids (brachy-dins A, B and C). Figure S2: FTIR spectra of BRA, empty F127, and BRA-loaded F127 micelles (LF-B500) were acquired in the solid state at room temperature. Table S1: HPLC-UV calibration curve for BRA fraction, Figure S3: Chromatogram of the LF-B500 formulation. The peak with the shortest retention time is attributed to BRA-A, the intermediary peak is related to BRA-B, and the peak with the longest retention time is attributed to BRA-C. The calculated concentration of BRA-A, BRA-B, and BRA-C were 55.89, 54.91 and 55.98 µg·mL^−1^, respectively, Figure S4: ζ potential of LF-B500 acquired by the DLS Technique in triplicate at 25 °C, Figure S5: In vitro cytotoxicity assay of F127 micelles against RAW 264.7 cell line murine and *Leishmania amazonensis* promastigotes for 0.125% (*w*/*v*) of F127 copolymer. The results correspond to (means ± SD) of individual samples tested in triplicate. (*) *p* < 0.05, compared to the positive controls (DMSO and pentamidine).
