# Supplementary material for: Design of Liquid Formulation Based on F127-Loaded Natural Dimeric Flavonoids as a New Perspective Treatment for Leishmaniasis
Source: Pharmaceutics. 2024 Feb 8;16(2):252. doi: 10.3390/pharmaceutics16020252 (PMC10891960; doi:10.3390/pharmaceutics16020252)
Supplement: Supplementary file 1 [file pharmaceutics-16-00252-s001.zip › pharmaceutics-2767464-supplementary.pdf]

SUPPORTING INFORMATION

Design of liquid formulation based on F127-loaded natural dimeric flavonoids as a new perspective treatment for leishmaniasis

Camila Silva da Costa [1], Estela Mesquita Marques [1], Jessyane Rodrigues do Nascimento [1,2], Victor Antônio Silva Lima [1], Ralph Santos-Oliveira [3,4], Aline Santana Figueredo [5], Caroline Martins de Jesus [5], Glécilla Colombelli de Souza Nunes [6], Clenilma Marques Brandão [7], Edson Tobias de Jesus [7], Mayara Coelho Sa [7], Auro Atsushi Tanaka [8], Gustavo Braga [8], Ana Caroline Ferreira Santos [8], Roberto Batista de Lima [8], Lucilene Amorim Silva [5], Luciana Magalhães Rebelo Alencar [9], Cláudia Quintino da Rocha [1] and Renato Sonchini Gonçalves [1,*]

[1] Laboratory of Chemistry of Natural Products, Department of Chemistry, Federal University of Maranhão, São Luís 65080-805, Brazil; cs.costa@discente.ufma.br (C.S.d.C.); estelamarques.adv@gmail.com (E.M.M.); jessyanernascimento@gmail.com (J.R.d.N.); vas.lima@discente.ufma.br (V.A.S.L.); rocha.claudia@ufma.br (C.Q.d.R.)

[2] Postgraduate Program in Chemistry, Institute of Chemistry, UNESP-Estadual University Paulista Júlio de Mesquita Filho, Araraquara 14800-060, Brazil

[3] Nuclear Engineering Institute, Brazilian Nuclear Energy Commission, Rio de Janeiro 21941-906, Brazil; presidenciaradiofarmacia@gmail.com

[4] Laboratory of Nanoradiopharmacy, Rio de Janeiro State University, Rio de Janeiro 23070-200, Brazil

[5] Laboratory of Immunophysiology, Center for Biological and Health Sciences, Federal University of Maranhão, São Luís 65080-805, Brazil; aline.sf@discente.ufma.br (A.S.F.); caroline.mj@discente.ufma.br (C.M.d.J.); lucilene.silva@ufma.br (L.A.S.)

[6] Research Nucleus in Pharmaceutical Sciences Program, State University of Maringá, Paraná 87020-900, Brazil; profglecillacolombelli@gmail.com

[7] Department of Chemistry, Federal Institute of Maranhão, São Luis 65075-441, Brazil; clenilma.brandao@ifma.edu.br (C.M.B.); tobiasedson@ifma.edu.br (E.T.d.J.); mayara.sa@ifma.edu.br (M.C.S.)

[8] Department of Chemistry, Federal University of Maranhão, São Luís 65080-805, Brazil; tanaka.auro@ufma.br (A.A.T.); gustavo.braga@ufma.br (G.B.); anacaroline.ufma@yahoo.com.br (A.C.F.S.); rb.lima@ufma.br (R.B.d.L.)

[9] Laboratory of Biophysics and Nanosystems, Department of Physics, Federal University of Maranhão, São Luís 65080-805, Brazil; luciana.alencar@ufma.br

* Correspondence: renato.sg@ufma.br; Tel.: +55-98-985149235

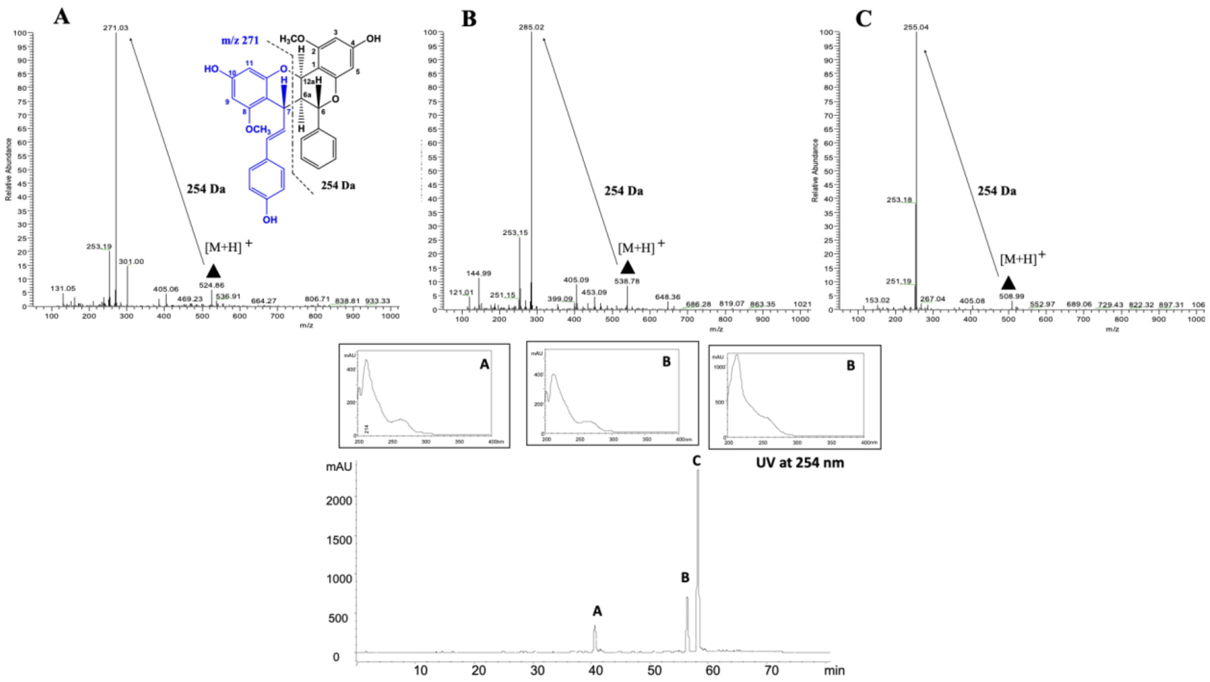

Figure S1: Chromatographic profile of the DCM fraction of *Arrabidaea brachypoda* roots (λ = 254 nm), UV spectra, and chemical structure of the three dimeric flavonoids (brachydins **A**–**C**).

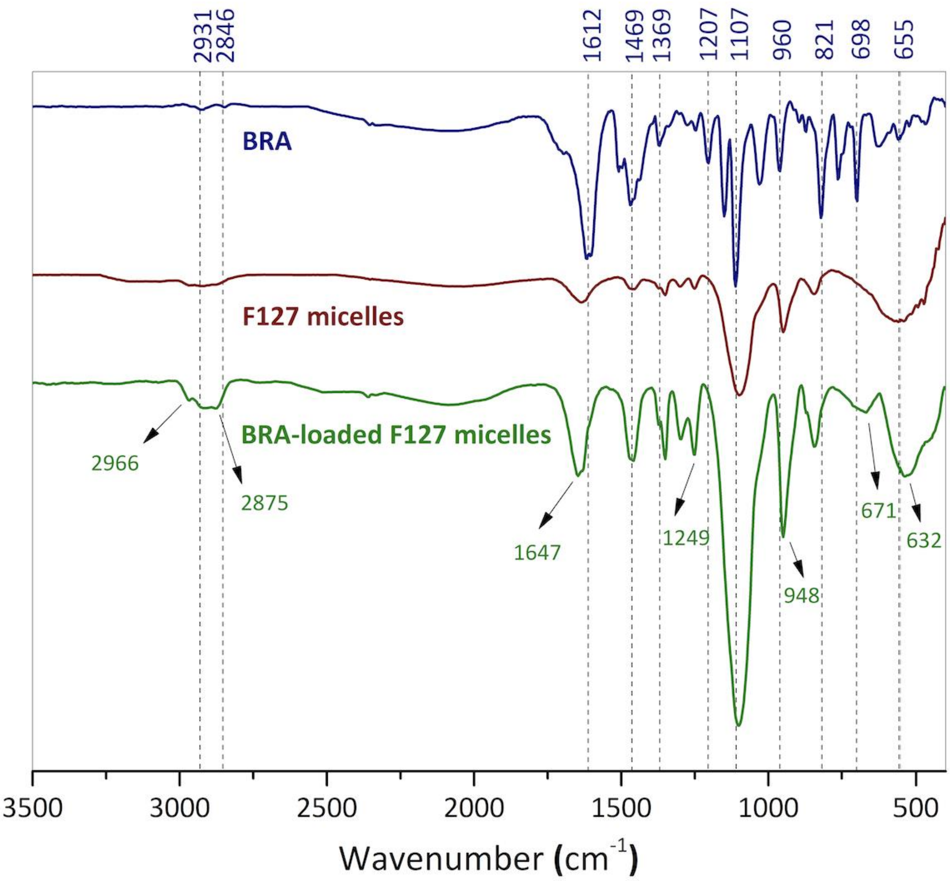

Figure S2. FTIR spectra of BRA, empty F127, and BRA-loaded F127 micelles (LF-B500) were acquired in the solid state at room temperature.

Table S1. HPLC-UV calibration curve for BRA fraction performed in triplicate.

Concentration (μg. mL^{-1})	Peak area ± SD	Concentration (μg. mL^{-1})	Peak area ± SD
10	222299 ± 12	60	980118 ± 32
20	410547 ± 25	70	1183534 ± 25
30	607817 ± 18	80	1264628 ± 31
40	748260 ± 21	90	1296007 ± 19
50	900200 ± 13	100	2635286 ± 11

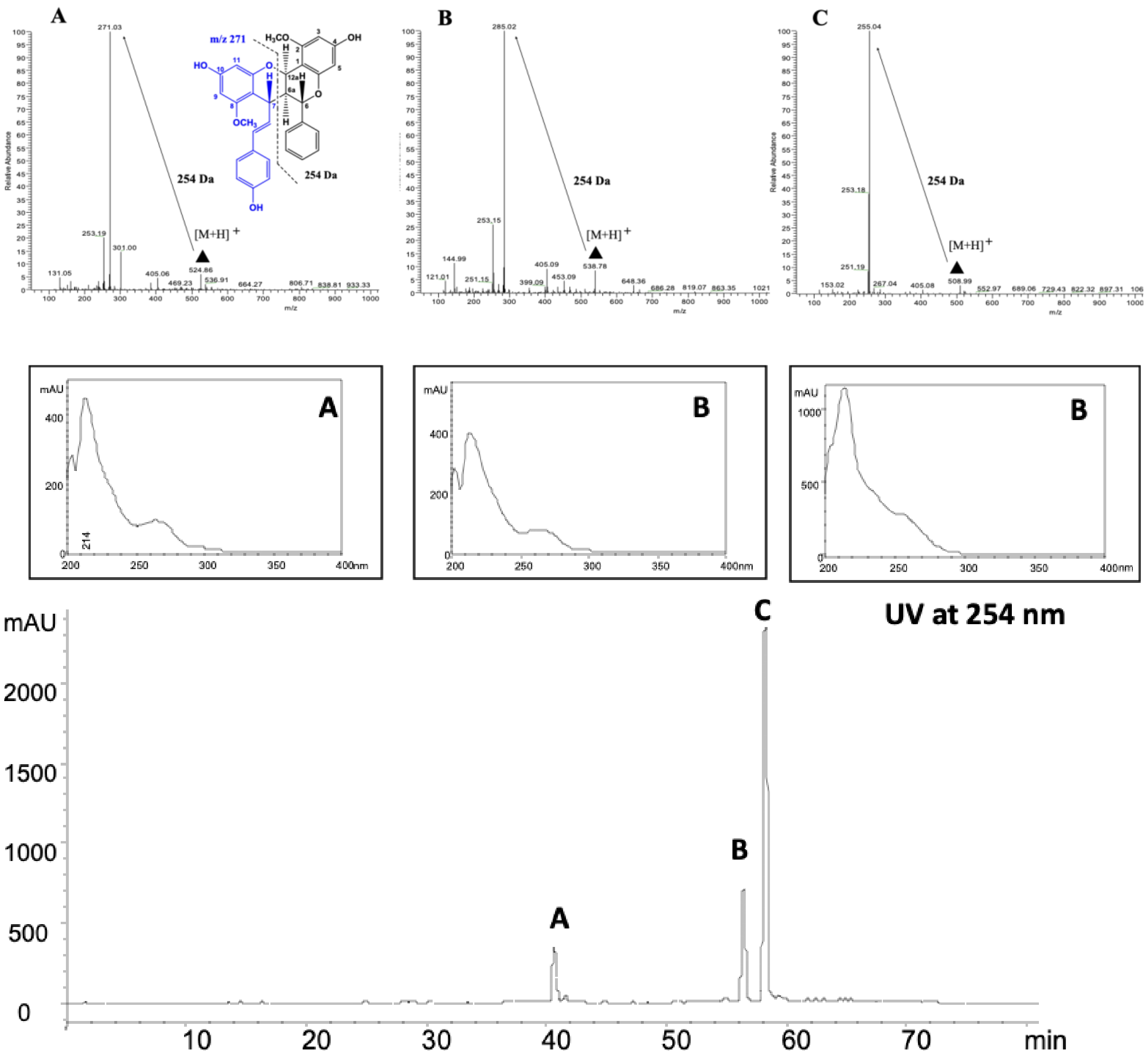

Figure S3. Chromatogram of the LFB formulation. The peak with the shortest retention time is attributed to BRA-A, the intermediary peak is related to BRA-B, and the peak with the longest retention time is attributed to BRA-C. The calculated concentration of BRA-A, BRA-B, and BRA-C were 55.89, 54.91 and 55.98 µg. mL^{-1} respectively.

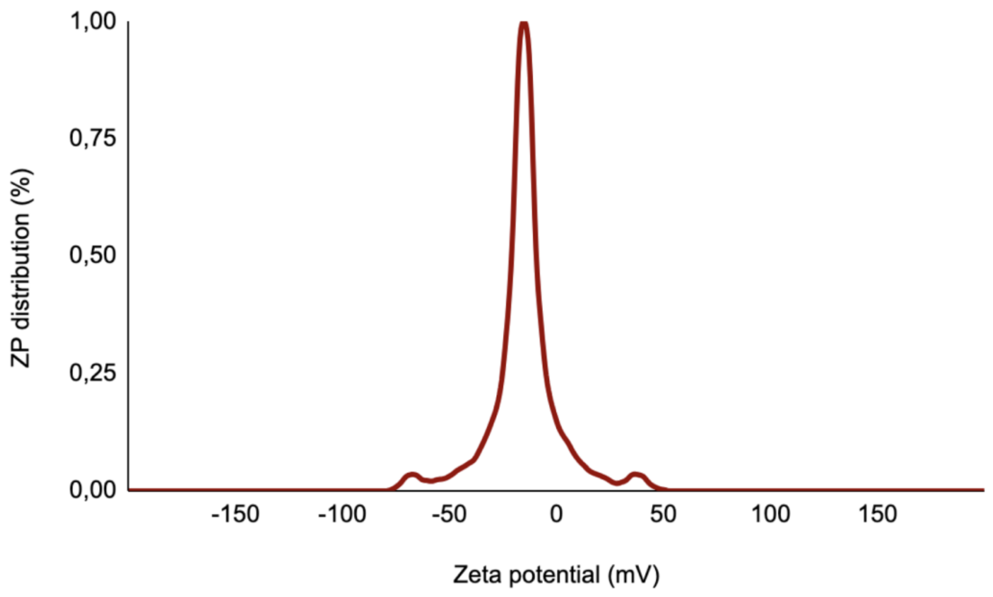

Figure S4. ζ potential of LF-B500 acquired by the DLS Technique in triplicate at 25° C

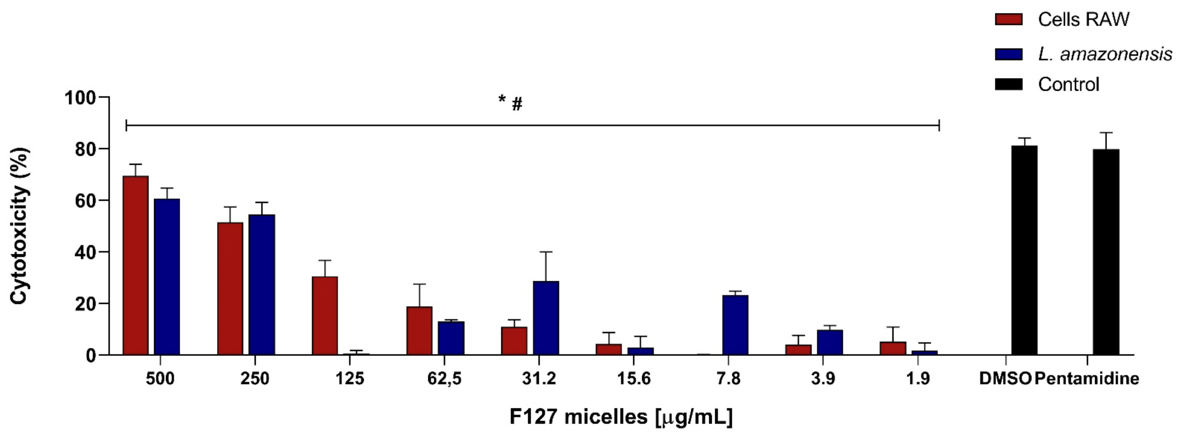

Figure S5. *In vitro* cytotoxicity assay of F127 micelles against RAW 264.7 cell line murine and *Leishmania amazonensis* promastigotes for 0.125% (w/v) of F127 copolymer. The results correspond to (means ± SD) of individual samples tested in triplicate. (*) $p<0.05$, compared to the positive controls (DMSO and pentamidine).
